# Delegated Regressor, A Robust Approach for Automated Anomaly Detection in the Soil Radon Time Series Data

**DOI:** 10.1038/s41598-020-59881-9

**Published:** 2020-02-20

**Authors:** Muhammad Rafique, Aleem Dad Khan Tareen, Adil Aslim Mir, Malik Sajjad Ahmed Nadeem, Khawaja M. Asim, Kimberlee Jane Kearfott

**Affiliations:** 1 Department of Physics Chehla Campus, University of Azad Jammu and Kashmir Muzaffarbad, 13100 Azad Kashmir, Pakistan; 2 Department of Computer Science and Information Technology, University of Azad Jammu and Kashmir, Muzaffarabad, 13100 Azad Kashmir, Pakistan; 3 Centre for Earthquake Studies, Islamabad, Pakistan; 4 University of Michigan, Department of Nuclear Engineering and Radiological Sciences, 48109-2104 Ann Arbor, MI USA; 5 GFZ German Research Center for Geosciences, Potsdam, Germany

**Keywords:** Computational science, Applied physics

## Abstract

We propose a new method based on the idea of delegating regressors for predicting the soil radon gas concentration (SRGC) and anomalies in radon or any other time series data. The proposed method is compared to different traditional boosting e.g., Extreme Gradient Boosting (EGB) and simple regression methods e.g., support vector regressors with linear kernel and radial kernel in terms of accurate predictions. R language has been used for the statistical analysis of radon time series (RTS) data. The results obtained show that the proposed methodology predicts SRGC more accurately when compared to different traditional boosting and regression methods. The best correlation is found between the actual and predicted radon concentration for window size of 2 i.e., two days before and after the start of seismic activities. RTS data was collected from 05 February 2017 to 16 February 2018, including 7 seismic events recorded during the study period. Findings of study show that the proposed methodology predicts the SRGC with more precision, for all the window sizes, by overlapping predicted with the actual radon time series concentrations.

## Introduction

During past few decades several studies have been carried out across the globe focusing on earthquake prediction based upon anomalous behavior of radon gas in atmosphere, soil and water. Many studies, since after first evidence of a correlation between radon in well water and earthquake (1966; *M* = 5.3) occurrence, reported by Ulomov and Mavashev in 1967 for the Tashkent earthquake, have recognized that anomalous behavior of radon in soil and groundwater can serve as a precursor for a forthcoming earthquake^1^. Sultankhodzhayev *et al*. in 1976 have reported the rise of the radon concentration in a spring before the Gazli earthquake (17 May 1976; *M* = *7.3*)^2^. A number of studies conducted in China reporting radon anomalies before strong earthquakes compelled scientists in the rest of the countries to carry out systematic investigation to probe possible link between radon anomalies and earthquake prediction^3,4^. Several studies have reported correlation between impending earthquakes with variability of radon gas in soil and ground water^5–23^.

Walia *et al*. 2005, have shown that micro-seismic events recorded along the Main Boundary Thrust (MBT) of N-W Himalaya in the grid (30–34°N, 74–78°E) have correlation with radon anomalies^24^. The same study revealed that 62% of micro-seismic events have correlation with the precursory nature of radon^24^. Their findings revealed, as reported in some other studies^20,23^, that radon anomalies are not only influenced by seismic events but also by meteorological parameters. Ramola *et al*. 2008, have reported spike-like and sharp peak anomalies in radon time series data before, during and after earthquake occurred in Garhwal Himalaya^15^.

Besides having a considerable number of research studies addressing radon anomalies serving for earthquake precursor based upon their experimental findings, yet there are some scientists who had produced thematic papers on radon as precursor for earthquake forecasting^25,26^.

Since anomalies in RTS data may arise due to multiple factors including seismic events, meteorological parameters, so forth. This leads to serious impediments in differentiating anomalies caused by seismic activities and those caused exclusively by environmental factors. Tareen *et al*. 2019 used same data set to identify the parameters, viz. environmental parameters, noise or seismic activity, influencing or triggering anomalies in radon time series data. Findings of that study showed that under meticulously characterized environments, on exclusion of noise contribution, seismic activity is responsible for anomalous behavior seen in current RTS data. Such situations can be handled using Machine Learning Methods (MLMs).

Machine learning has been successfully applied to many problems in the environmental sciences^27^. With MLMs, a model for the prediction of radon concentration can be built, taking into account various environmental parameters (e.g., barometric pressure, rainfall, and air and soil temperature). Such models can subsequently be used to identify radon anomalies triggered by seismic events. The application of artificial neural networks^28–30^, regression and model trees^31–34^ and different other methods^32,35^ have proven to be useful for extracting radon anomalies caused by seismic events.

Diagonal Linear Discriminant Analysis- DLDA^36^, *k*-Nearest Neighbors-*k*NN^37^, Support Vector Machine^38^ and Random Forest^39^ have been employed for classification and regression purposes. These methods have applications in decision support systems^40–42^ and earthquake prediction studies^33,43–45^. Analyses of the radon data from three stations in the Krsko basin, Slovenia^33^, showed that model trees outperformed other regression methods. Negarestani *et al*., 2002, experimented layered neural network (LNN) to estimate the radon concentration in soil related to the environmental parameters that can find any functional relationship between the radon concentration and the environmental parameters^28^. Singh *et al*., 1999, observed the significant increases in radon concentration of groundwater and water level which are correlated to the seismic events which occurred in Northern India during the period of study^46^.

Freund and Schapire 1998, proposed the well-known AdaBoost.M1 (also known as Discrete Adaboost) algorithm^47^. Friedman *et al*.^48^ worked on boosting and developed gradient boosting algorithm, which uses machine learning techniques to make weak classifiers usually decision tress and then make the final prediction, is based on the aggregate of this weak classifier. In 2000, He established connections of Adaboost.M1 algorithm to statistical concepts such as loss functions, additive modeling, and logistic regression. The step of taking the random sampling in boosting is motivated by Breiman’s bagging procedure that makes the nature of boosting to be stochastic. In addition, it develops the idea of delegating classifiers in a systematic way by delegating the difficult or uncertain predictions to other, possibly more specialized classifiers. On the other hand, Ferri *et al*., 2004, also presented an iterated scenario involving an arbitrary number of chained classifiers^26^.

In this study we propose a new method based on delegating classifiers^26^ for predicting the radon concentration and anomalies in soil radon time series data by delegating the samples to the next lower level that do not meet the desired threshold e.g., uncertain predictions. The proposed methodology has foundations regarding classification task by keeping the power of delegation in classification. For analysis purpose, RTS data was obtained for the period from 05 February 2017 to 16 February 2018 including 7 seismic activities recorded during this period. The proposed method is compared to different traditional boosting (Extreme Gradient Boosting) and simple regression methods (support vector machines with linear kernel support vector machines with radial kernel) based on how much they accurately predict the radon concentration. The extreme gradient Boosting method is the most popular and extensively used ensemble approach that has had been successfully used for the regression problems and also Support Vector Machine technique (with linear and radial kernels) which is also the most popular method for regression problems. The results obtained depicts that the proposed methodology predicts more accurately the RTM 1688-2 measured RTS data when compared to different traditional boosting and regression methods.

## Materials and Methods

### Location and instrumentation

Current study is performed in Muzaffarabad, a city in Pakistani territory of Kashmir. A radon station, for the continuous measurement of radon time series data, was installed in highly active seismic zone. RTM 1688-2, (SARAD RTM 1688-2, Nuclear Instruments, Germany) was installed at the fault line passing beneath the Chehla with latitude 34.39621 and longitude 73.47347. The location of radon monitoring station lies within 150 km of the Centre of the strongest earthquake in the region since 1900. Packer probe was placed with already digged borehole and sealed to avoid ambient air contact. Than Packer probe was connected to the RTM 1688-2, (SARAD RTM 1688-2, Nuclear Instruments, Germany), placed within a 1 meter of the soil surface.

The RTM 1688-2 measures the ^222^Rn concentration in slow i.e., contributions from the disintegrations of both ^218^Po and ^214^Po, and Fast modes for which only ^218^Po decay events are counted. The RTM 1688-2 device measures humidity (0 to 100%), temperature (−20 to 40 °C) and barometric pressure (800 to 1200 mbar). We have operated the instrument in slow mode for measurement of radon gas in current study. RTS data were collected in 40 min intervals with 36 readings per day spanning over a period of one year.

### Proposed methodology

**Figure 1:**
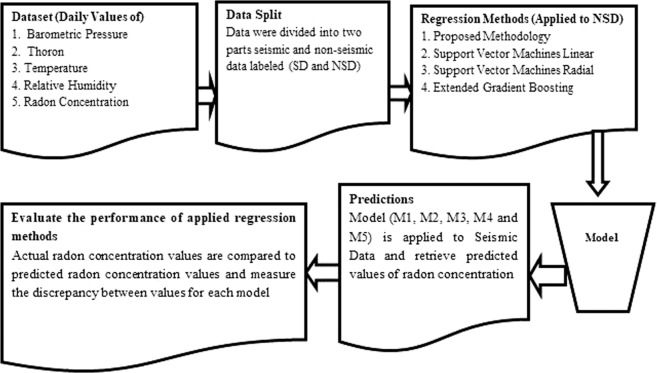
Simulation Plan for radon anomaly detection using different machine learning methods.

Complete simulation plan for radon anomaly detection using different machine learning methods is shown in Fig. 1.

Since radon concentration is a numeric variable, we have approached the task of predicting radon concentration from meteorological data using regression (or function approximation) methods. In order to predict the radon concentration at different periods of time before and after the seismic activity, the dataset is divided into two parts i.e. seismic and non- seismic radon data. For each window size the seismic radon data comprises of the days before and after the seismic events viz. window size of 1 means 1 day before and after the seismic event. Algorithm developed for proposed methodology is given as;

**Algorithm 1:**
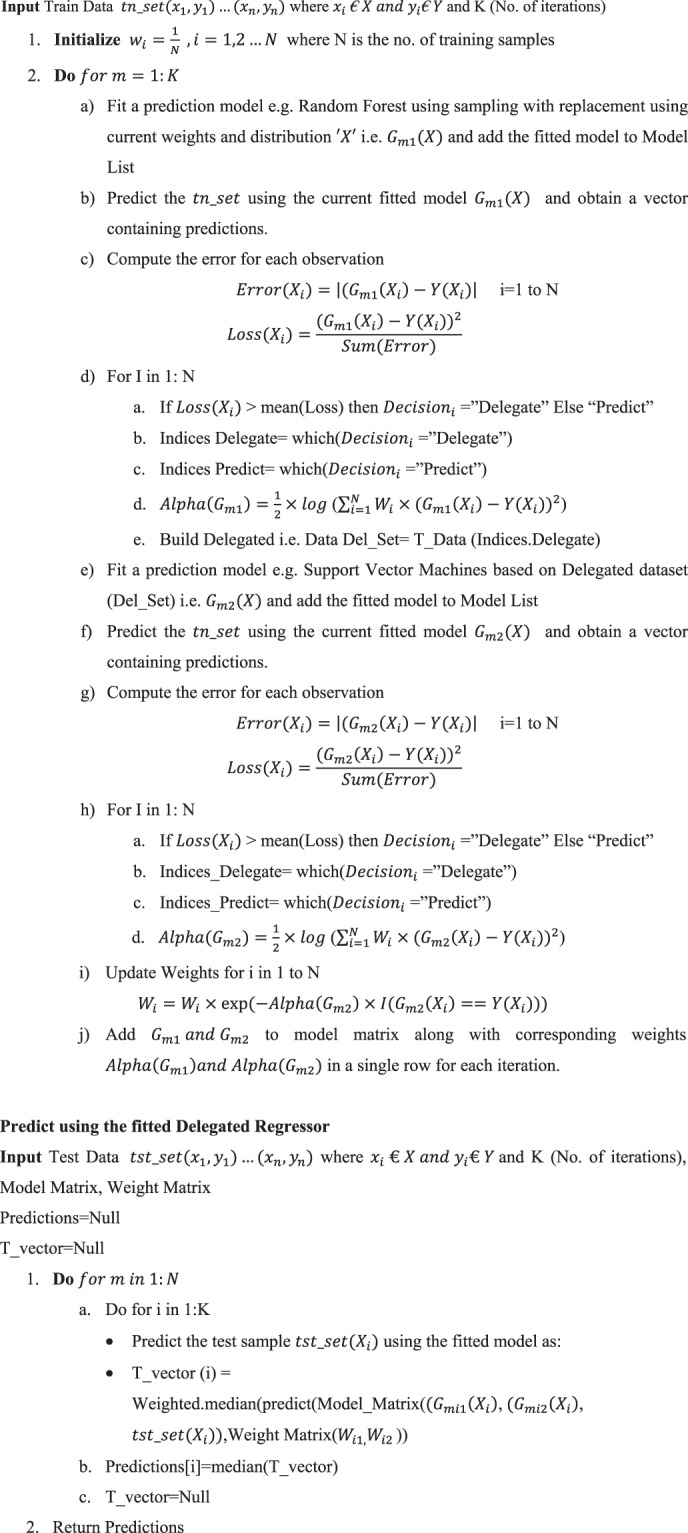
Proposed Delegated Boosting for Regression.

### Return predictions

The total number of samples in the dataset are 13456; including 7 seismic activities. The data has been divided into two parts: seismic data, containing anomalies, and treated as test data while non-seismic, without anomalies, and is taken as training data. The number of samples in the training and test data varies with respect to window size. With the increase in window size, the number of samples in the test data starts increasing as compared to training data which decreases. On increasing the window size, i.e. days before and after the seismic activity, the sample from non-seismic training data is added to seismic data (i.e. test data).

The predictive performance of the regression methods was determined using root mean squared error (RMSE). The RMSE measures the discrepancy between measured and predicted values of radon concentration. Smaller RMSE values indicate lower incongruities. There are other metrics in order to measure the error for predictions e.g. MSE (mean squared error) which is the most simpler and commonly used metric for regression evaluation tasks but the problem in this metric is that if we make only a single prediction very bad, it makes the error even worst because of squaring the distance between actual and predicted values and skews the metric in the direction of overvaluing the computed model’s badness. In order to make errors to meet the scale of targets, a square root is introduced on MSE but travelling along the RMSE gradient is same as travelling along MSE gradient but at a different flowing rate. Moreover, in the literature authors also used RMSE as a metric for estimation of error.

To test the hypothesis for the predictability of radon concentration in periods with and without seismic activities, the following procedure was applied. First, the value of the class—daily radon concentration; and the values of attributes—barometric pressure, thoron, temperature and relative humidity was selected. Second, this data set was split into two parts. In the first part (labeled SA), data for the periods with seismic activity were included, i.e., periods of 12 days before and after an earthquake. Data for the remaining days were included in the second part, belonging to the seismically non-active periods (labeled non-SA). We have applied the methodology by utilizing the proposed and traditional methods, we trained the models on non-seismically active data part and obtained each respective model. Moreover, from each model we have predicted the seismically active data part and obtained the predicted radon concentration. Finally, we estimated the error regarding predicted and actual values. The performance of the model depends upon how much the actual and predicted values of radon are closed to each other.

## Machine Learning Prediction Methods

### Extreme gradient boosting

XGBoost (Extreme Gradient Boosting) has become one of the most popular machine learning algorithms for classification and prediction problems^49^. Gradient Boosting was developed as a generalization of AdaBoost by observing gradient search of AdaBoost in decision tree space against a particular cost function^50^. The innovation of Gradient Boosting39,51 was the observation that can use different cost functions, some of which were more suitable to the domain than the one that was implicitly used in AdaBoost.

Gradient Boosting was however overwhelmed by a lot of ad-hoc parameters to control the growth of the decision trees in the algorithm^51^. XGBoost^52^ was developed to put this on a more formal footing. In XGBoost the size of the tree and the magnitude of the weights are controlled by standard regularization parameters. This leads to a ‘mostly’ parameter-free optimization routine. In theory that is, as in practice a plethora of parameters are used, still to control the size and shape of the trees. Regularization did however proved to be very powerful and made the algorithm much more robust.

Real extreme gradient boosting is better regularized model formalization of Gradient Boosting that gives better performance to control the over-fitting problem. Therefore, it helps to reduce over fitting regarding training data. Its roots begins from the implementation of gradient boosting machines but now because of its efficiency and better performance, it is now associated to a more extensive collection of tools under the umbrella of the distributed machine learning community^53^.

### Support vector machine (linear and polynomial)

Vladimir Vapnik, 1979 and his co-workers introduced a Support Vector Machines (SVMs)^54^. SVMs is a well-known method used for classification and regression problems both for linear and non-linear types of data^39^. By utilizing non-linear methods it refurbishes the data in to high dimensions. The main theme of support vector machines is to find the finest linear decision boundary to discriminate different categories. Moreover, the fastest version of SVMs can take much time for training of data but in result, returns the more accurate classifications or prediction. SVMs had been successfully applied for different regression problems^55–57^ by maintaining the entire main features i.e. maximal margin that makes algorithm ability to differentiate itself. Support Vector Regression encompasses the same foundations as we used as the SVMs for classification tasks only having a few little differences. As we know that in regression problems, the output is the real values that make it difficult to predict the information at hand because of having inestimable possibilities. In order to make it predictable, epsilon (margin of tolerance) is approximated to the SVM that is previously asked from the problem. On the other hand, in order to minimize the error rate, hyperplane are individualized based on how much they maximize the margin and also taking in to account that part of error is tolerated.

As like SVM works for classification problems, regression is also done by providing a loss function that tolerates errors within a certain margin^58^. Moreover, that $\varepsilon$-band contain points called as support vectors^59^.

1 $$\begin{array}{rcl}L(y,f(x,w)) & = & \{\begin{array}{l}0\,if\,|y-f(x,w)| < \varepsilon \\ |y-f(x,w)|-\varepsilon ,\,otherwise\end{array}\end{array}$$

where $f(x,w)$ is a linear model function;

2 $$f(x,w)={\sum }_{j=1}^{m}{w}_{j}{g}_{j}(x)+b$$

with ${g}_{j}(x)$ as a set of transformation functions that are aimed to map input x to m-dimensional feature space. b is the bias term, which can be ignored when the data is preprocessed to be zero-mean. Basic representations of SVM and SVR with slacked variables are shown inFigs. 2 and 3 respectively^60,61^.

**Figure 2:**
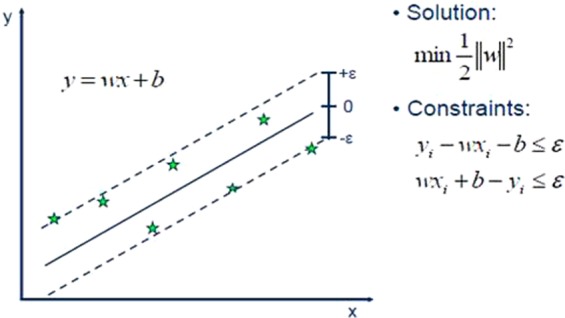
Basic representation of Support Vector Machine for regression^60^.

**Figure 3:**
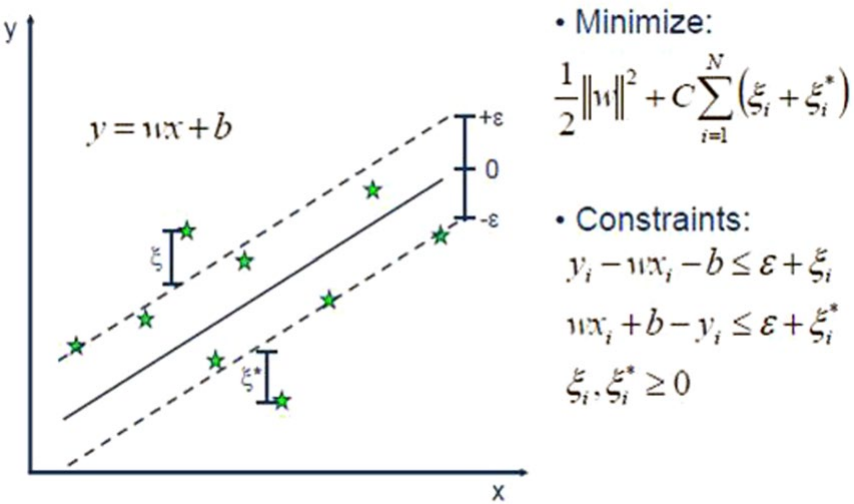
Support Vector Regressor with slacked variable^60^.

## Results and Discussion

This section presents the results to evaluate the performance of the proposed methodology, delegated regressor, in comparison with other regression methods in terms of different performance measure and accuracy for predicting radon concentration during the seismic periods of windows ranging for size 1 to 12.

**Figure 4:**
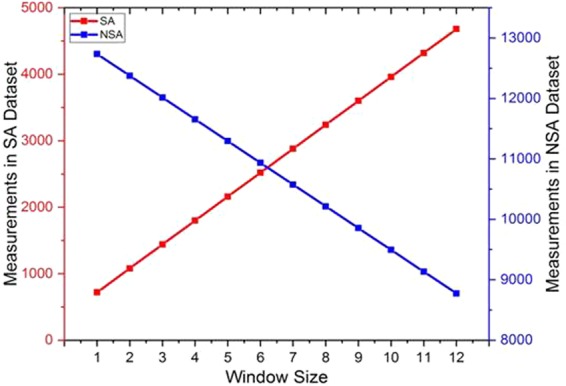
Number of measurements in seismic and non-seismic dataset with respect to window sizes ranging from 1 to 12.

It is noted that upon increasing the window size, the error in prediction of the radon concentration increases. This is due to decrease in training data samples caused by splitting of data during each window operation. As the window size increases the radon seismic data samples grows at the cost of decreasing number of instances for training of MLs, as shown in Fig. 4.

Figure 4 shows the number of measurements after splitting of whole data in to seismic activity (SA) and non-seismic activity (NSA) datasets. Non-seismic data set are those days for which no seismic activity have been observed. And these non-seismic data sets dynamically change with respect to window size.

**Figure 5:**
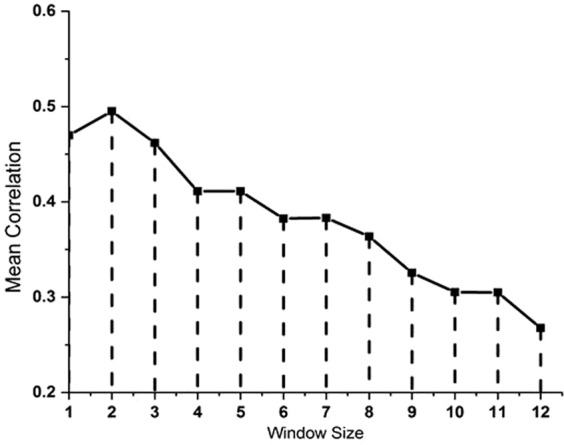
Mean correlation of actual and predicted radon concentration using different regression methods.

Figure 5 shows the mean linear correlation regarding different regression methods for the actual and predicted radon concentrations in soil. With the fact that number of measurements gets decreased with the increase in window size, the correlation between the actual and predicted radon concentration in soil also decreases. The best correlation found between the actual and predicted radon concentration is at window size of 2. This demonstrates those two days before and after the seismic activities are very important in order to accurately predict the radon anomaly which is an earthquake precursor.

## Comparison of Delegated Regressor with Other Traditional Regressors

Results obtained from proposed delegated regressor algorithm have been compared with other regression methods for the prediction of radon concentration in soil. The dataset distribution regarding SA and NSA was carried out in such a way that SA data contains varying number of days ranging from 1 to 12 before and after the seismic activity.

Table 1 presents the error rate of proposed delegated and other regression methods for the prediction of radon concentration in soil. On the same dataset, regarding same window size extracted data, the proposed delegated Regressor outperforms then other regression methods having minimum RMSE. Experimental data for the period from 05 February 2017 to 16 February 2018 is used for computer experimentations and simulation purpose.

**Table 1:** Root mean squared error (RMSE) of different regression methods for prediction of radon concentration in soil with respect to different window sizes.

Win Size	Extreme Gradient Boosting (XGBoost)	Support Vector Machine Linear (SVML)	Support Vector Machine Radial (SVMR)	Delegated Regressor Method (DRM)
1	2505.088	6108.028	4826.279	1809.784
2	2473.006	6149.988	4977.869	1806
3	2416.071	6225.425	5318.782	2017.899
4	2518.619	6365.508	6527.553	1927.861
5	2593.956	6531.951	6699.957	1731.9
6	2670.358	6745.238	7109.096	2479.699
7	2761.739	6945.34	7704.349	2200.264
8	3033.962	7150.594	9223.404	1991.526
9	3135.076	7223.843	9424.501	2269.731
10	3138.239	7396.725	9659.676	2291.885
11	3596.169	7568.502	10229.27	2300.619
12	3757.667	7618.878	10881.65	2667.437

The maximum error rate for the proposed methodology (2667.437) is appreciably smaller when compared to other Regressor methods (XGBoost, SVML and SVMR) having maximum error rates of 3757.667, 7618.878 and 10881.65 respectively.

**Figure 6:**
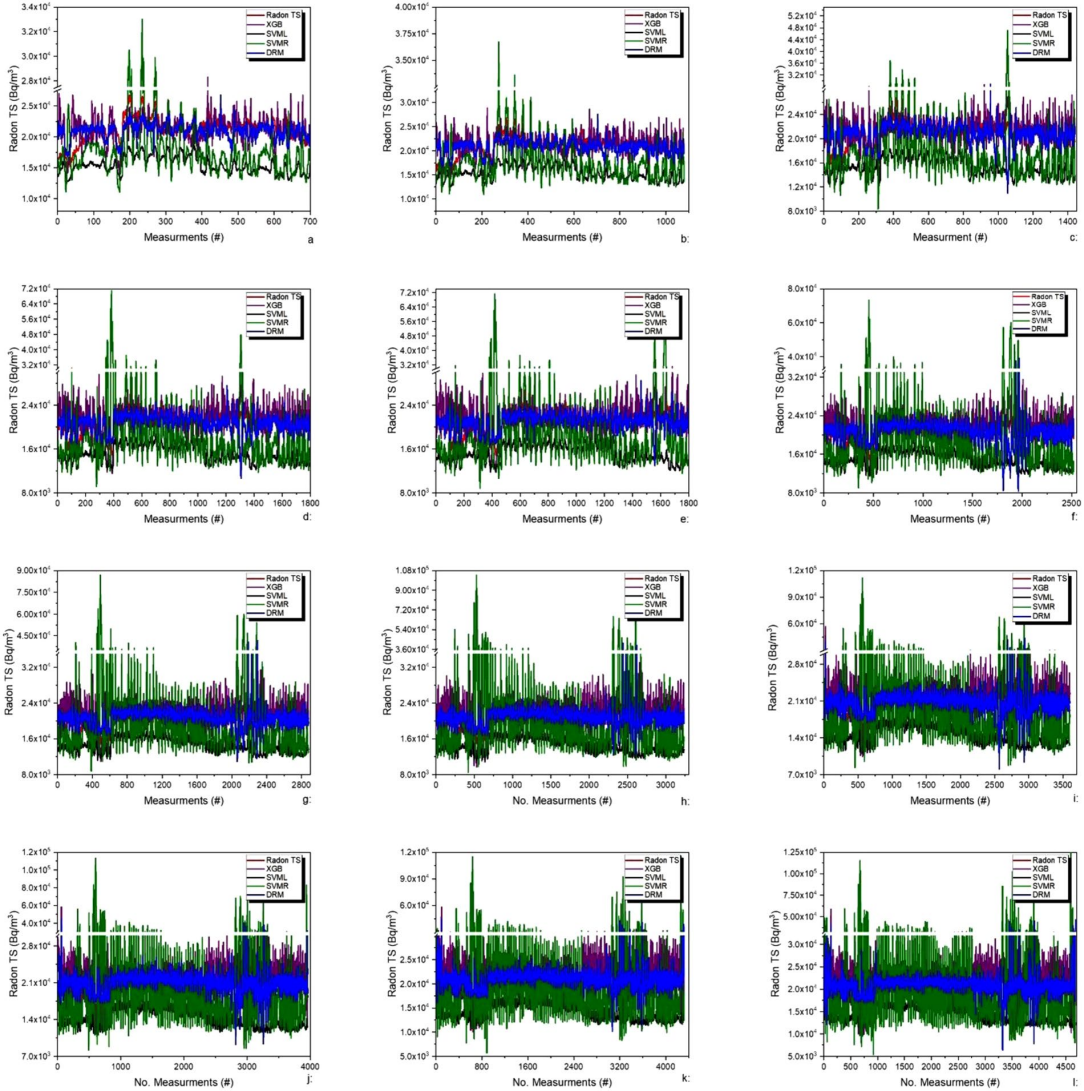
(**a**–**l**) Represents radon concentration for 1 through 12 days before and after earthquake with red lines actually showing the earthquake with its magnitude.

For the window 1, one day before and after earthquake, Fig. 6(a) shows the actual and predicted RTS data using delegated and other regression methods. On the X axis we have a number of measurements and Y axis represents the radon concentration. The Fig. 6(a) shows that delegated Regressor predicts more accurately than other regression methods by overlapping real time RTS data with better results than other regression methods. Proposed delegated Regressor Method (PDRM) shows that RMSE is less for PDRM (1809.784) when compared to other regression methods viz., XGBoost (2505.088), SVML (6108.028) and SVMR (4826.279) for the prediction of radon concentration in soil. All these evaluation criteria show that the PDRM outperforms other regression methods. Figure 6a shows anomalies in real RTS data can be predicted well by PDRM. Trend in series generated by PDRM follows trend of real RTS.

The results on same pattern were observed for the windows 2 through 12. For the 2^nd^ window (see Fig. 6b), two (02) days before and after earthquake, we have obtained lowest value of RMSE as compared to all other windows. PDRM showed that RMSE is less for PDRM (1806) when compared to other regression methods viz., XGBoost (2473.006), SVML (6149.988) and SVMR (4977.869) for the prediction of radon concentration in soil.

RMSE computed from all MLs techniques and PDRM, for the window 3, shows that RMSE is less for PDRM (2017.899) when compared to other regression methods viz., XGBoost (2416.071), SVML (6225.425) and SVMR (5318.782) for the prediction of radon concentration in soil (see Fig. 6c). The results for all other windows 4 through 12 are shown in Table 1. All these statistics shows that the PDRM outperforms other regression methods for the window size 1 through 12. Almost for all windows, 1 through 12, PDRM trend follows RTS real time trend (see Fig. 6a–l).

**Figure 7:**
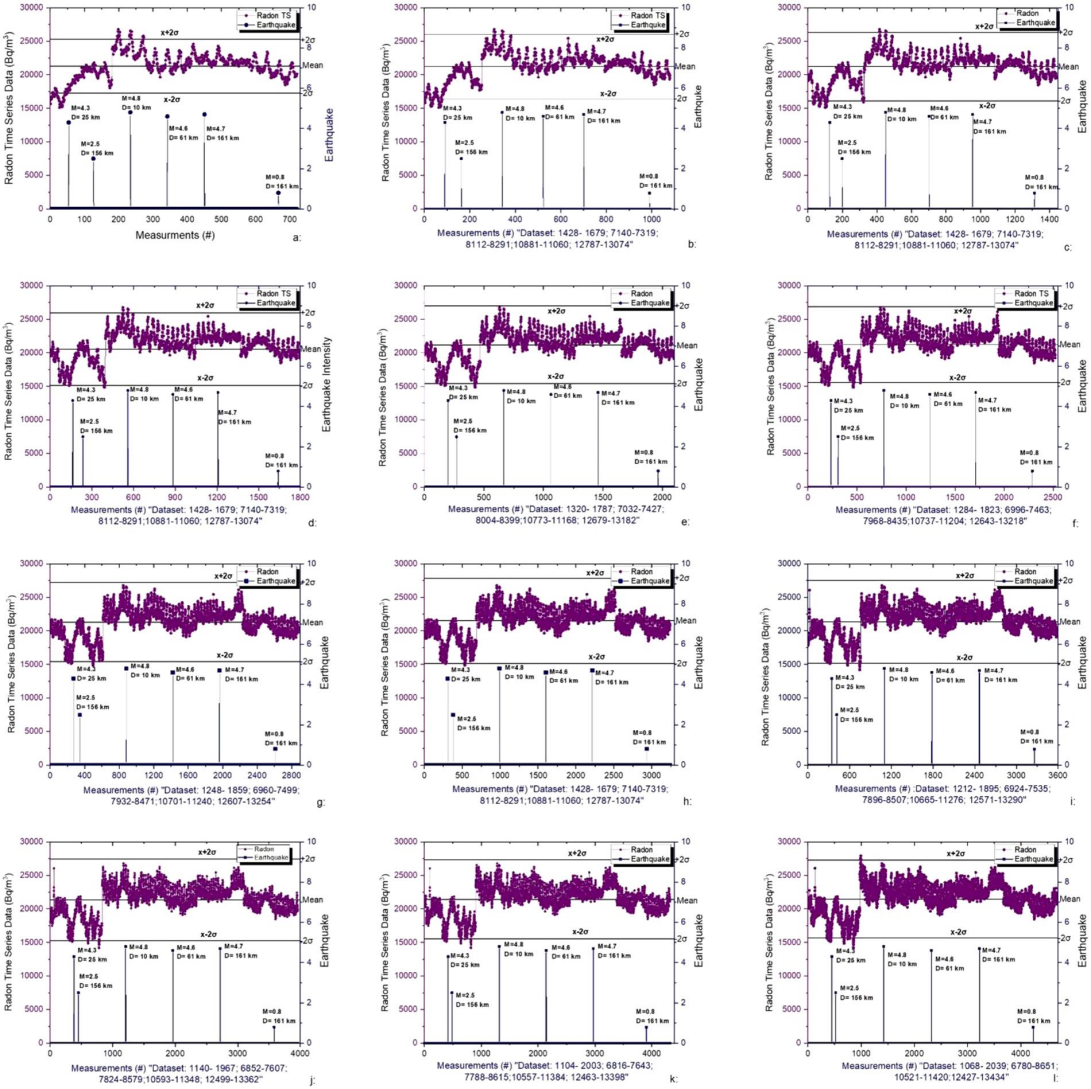
(**a**–**l**) Actual and predicted radon concentration using delegated and other regression methods using window size of one through 12 days.

Figure 7(a) through 7(l) shows the RTS data recorded from one through twelve days before and after each earthquake struck in the area of study. Vertical lines, olive green, show the earthquake with its magnitude (see Fig. 7a–l).

Radon anomalies leading to possible earthquake have been predicted using the method of standard deviation (see Fig. 7a–l). To differentiate between anomalies caused by environmental data from seismic events we have chosen period for anomaly for which radon concentration is deviated by more than ±2σ^61–64^. For the window 1 four spikes, with radon concentration 26763, 26539, 25773 and 15127 Bq/m^3^, in RTS data were recorded for which radon concentration deviated by more than ±2σ. Anomaly, at 15127 Bq/m^3^, in RTS data was followed by an earthquake, after one day, with magnitude 4.3 at the depth of 25 km with Lat (34.91°N) and Long (72.94°E) on 21^st^ of March 2017 (see Fig. 7a). Spike observed at 26763 Bq/m^3^ was followed by another earthquake after one day with magnitude 4.8 at richter scale. Earthquake occurred at the depth of 10 km with Lat (33.81°N) and Long (73.19E°E) on 27^th^ of August 2017. Another spike in radon concentration 25773 Bq/m^3^ was followed by earthquake of magnitude 4.6 after one day. This earthquake occurred on 23^rd^ of September 2017 at the depth of 61 km with Lat N(35.48 N) and Long E(73.01 E). For the window one it was observed that the three earthquakes were struck soon after observing radon anomaly before one day.

For the window 2, RTS deviated −2σ pattern during measurement 1490 and 1497 with radon soil concentrations 15126 and 15197 Bq/m^3^ respectively. These anomalies in radon concentrations were followed by an earthquake of magnitude 4.3 during measurement number 1510. Two more anomalies in RTS data were observed, exceeding +2σ, during measurement numbers 7196 and 7231, followed by earthquake of magnitude 4.8 on richter scale. Almost same pattern was observed for rest of windows 3 through 12.

## Conclusion

This study proposed a new approach for regression based on delegating classifiers. The idea behind the proposed method is that the examples having predictions not lie on a reliable threshold gets delegated to the next lower level with the hope that the regressor at the next level will become more specialized to predict these delegated examples. Moreover, we have also compared the proposed delegated regressor to other machine learning methods such as XGBoost, SVML and SMR to predict radon concentration in soil gas from measured environmental data, i.e. relative humidity, temperature and pressure. From the statistics above we have concluded that the proposed methodology predicts the radon concentration with more precision for all the window sizes by overlapping the actual radon concentration and all of the 6 earthquakes. Our measurements are still in progress and further analyses will be carried out over longer number of measurements.

## Data Availability

All data included in the manuscript are available upon request by contacting with the corresponding author.
